# On some squat lobsters from India (Decapoda, Anomura, Munididae), with description of a new species of *Paramunida* Baba, 1988

**DOI:** 10.3897/zookeys.965.55213

**Published:** 2020-09-03

**Authors:** Enrique Macpherson, Tin-Yam Chan, Appukuttannair Biju Kumar, Paula C. Rodríguez-Flores

**Affiliations:** 1 Centre d’Estudis Avançats de Blanes (CEAB-CSIC), C. acc. Cala Sant Francesc 14 17300 Blanes, Girona, Spain Centre d’Estudis Avançats de Blanes Blanes Spain; 2 Institute of Marine Biology and Center of Excellence for the Oceans, National Taiwan Ocean University, Keelung 20224, Taiwan, ROC National Taiwan Ocean University Keelung Taiwan; 3 Department of Aquatic Biology and Fisheries, University of Kerala, Thiruvananthapuram 695581, Kerala, India Univeristy of Kerala Kerala India

**Keywords:** *
Agononida
*, Indian Ocean, integrative taxonomy, molecular characters, morphology, *
Munida
*, new record

## Abstract

Squat lobster specimens belonging to the family Munididae were recently collected along the southwestern coast of the mainland of India and in the Andaman Islands. The specimens belong to two known species, *Agononida prolixa* (Alcock, 1894) and *Munida compacta* Macpherson, 1997, and a new species, *Paramunida bineeshi***sp. nov**. We here redescribe *A. prolixa* and describe and figure the new species. *Munida compacta* is newly recorded from India, and we figure the live coloration. In addition, molecular and phylogenetic analyses of two mitochondrial markers (16S rRNA and COI) revealed the phylogenetic relationships of *M. compacta* and *P. bineeshi***sp. nov.** with their most closely related congeners. The genetic similarity among the individuals of *M. compacta* from different locations is also addressed.

## Introduction

Squat lobsters are a very diverse and abundant group of anomuran decapods that are distributed throughout the world (Schnabel et al. 2011). A high level of squat lobster diversity is found in the western-central Pacific Ocean, particularly in the Solomon-Vanuatu-New Caledonia region, the Coral Sea, the Indo-Malay-Philippine archipelago, and French Polynesia (Macpherson et al. 2010; Schnabel et al. 2011; Rodríguez-Flores et al. 2019a).

During the nineteenth century, several expeditions were carried out in the Indian Ocean by the Indian survey steamer ‘Investigator’. The results of these expeditions – in which abundant material was gathered and many new species discovered – had been published by several workers, e.g., Wood-Mason and Alcock (1891), Alcock (1894, 1901), Alcock and Anderson (1894, 1899a, 1899b), Anderson (1896), McArdle (1901), MacGilchrist (1905), and Lloyd (1907). Of the many new species described by these authors, most were from the Bay of Bengal and the Arabian Sea. Deep-sea expeditions by the German vessel ‘Valdivia’ also gathered extensive material, including five new species (Doflein and Balss 1913). In addition to these expeditions, other relatively recent investigations in the Indian waters have revealed many new findings on squat lobsters, including descriptions of new species (e.g., George and Rao 1966; Rao 1974; Tirmizi and Javed 1993; Thirumilu 2011; Vaitheeswaran and Venkataramani 2012; Vaitheeswaran 2014, 2015, 2017; Komai et al. 2019). Although the seas around India are considered to be a moderately rich biogeographic region (cf. Spalding et al. 2007; Schnabel et al. 2011), the current diversity of squat lobsters from the region is not very high, and more research is needed to improve our knowledge on the group in this interesting oceanic province.

Recently, we collected specimens from India that constitute three species belonging to the genera *Agononida* Baba & de Saint Laurent, 1996, *Munida* Leach, 1820, and *Paramunida* Baba, 1988, all from the family Munididae Ahyong, Baba, Macpherson & Poore, 2010. Among our material, the lone specimen of *Paramunida* from the Andaman Islands is described herein as a new species, *Paramunida bineeshi* sp. nov. *Agononida prolixa* (Alcock, 1894) is redescribed based on a male specimen from the Andaman Islands. Several specimens of *Munida compacta* Macpherson, 1997 were collected from Kerala (southwestern coast of the Indian mainland), and the species newly recorded in Indian waters. The live colouration of *M. compacta* is described and figured here. We also conducted molecular and phylogenetic analyses using two mitochondrial markers, cytochrome oxidase subunit I (COI) and 16S rRNA (16S), for *M. compacta* and *P. bineeshi* sp. nov. to determine the phylogenetic relationships with their most closely related congeners. Furthermore, we noticed the genetic similarity among the individuals of *M. compacta* from different localities.

## Material and methods

The material, including the holotype of the new species, is located in the Department of Aquatic Biology and Fisheries, University of Kerala, Thiruvananthapuram, Kerala, India (**DABFUK**). The specimens from the Andaman Islands were collected by K.K. Bineesh of the Andaman and Nicobar Regional Centre, Zoological Survey of India. The terminology and measurements follow Baba et al. (2009, 2011). The size of the specimens is indicated by the postorbital carapace length (**CL**), measured along the midline from the base of the rostrum to the posterior margin of the carapace. The rostrum was measured from its base, situated at the level of the orbit, to the distal tip. Measurements of appendages were taken in dorsal (pereopod 1), lateral (antennule, pereopods 2–4) and ventral (antenna) midlines. Abbreviations used are: **Mxp3**, maxilliped 3; **P1–4**, pereopods 1–4.

### Molecular analysis

Tissue, taken from one of the pereopods, was used to extract genomic DNA with the DNeasy (Qiagen) kit following manufacturer’s protocol. A prior digestion of the sample was performed during 18–24 hours, and RNase was included before the extraction. Partial sequences of the mitochondrial COI and 16S partial genes were amplified by polymerase chain reaction (PCR) using combinations of the following primers: tenuiCOIFwint/ tenuiCOIRev1int/ tenuiCOIRev2int (Rodríguez-Flores et al. 2019a), LCO1490 (Folmer et al. 1994), COI-H (Machordom et al. 2003) for COI; and 16SAR and 16SBR (Palumbi et al. 1991) for 16S markers. The amplified fragments were purified using ExoSAP-IT (Affymetrix). Sequencing of both strands was performed using BigDye Terminator in an ABI 3730 genetic analyzer in the SECUGEN service (Madrid, Spain). Forward and reverse DNA sequences were obtained for each specimen, which were checked and assembled using the program Sequencher 4.8 (Gene Code Corporation). Multiple alignment for the 16S marker was carried out using MAFFT (Katoh et al. 2002) with a posterior manual correction in AliView alignment editor (Larsson 2014).

Genetic distances between species were estimated using uncorrected divergences (*p*) calculated using PAUP v. 4.0 (build 167) (Swofford 2004). All the obtained sequences were submitted to GenBank [Accession numbers: *Munida compacta* Macpherson, 1997 (MT829201, MT827008) from Papua, (MT829202-3, MT827009-10) from Indian waters, *Paramunida bineeshi* sp. nov., holotype (MT829200, MT828867)].

Bayesian phylogenetic analyses were performed with MrBayes v. 3. 2. 1 (Huelsenbeck and Ronquist 2001) using matrices with concatenated COI and 16S for some closely related species of *Munida* and *Paramunida* separately. The sequences of the related species were obtained from GenBank, with the following accession numbers: KY230467-8 (holotype), KY230451-2 (paratype) (*Munida benguela* de Saint-Laurent & Macpherson, 1988); AY351115, AY350945 (*Munida congesta* Macpherson, 2000); AY351152, AY350985 (*Munida pagesi* Macpherson, 1994); AF283885-6, AY351160-1 (*Munida rhodonia* Macpherson, 1994); AY350994, AY351162 (*Munida rosula* Macpherson, 1994); AF283887, AY351163 (*Munida rubridigitalis* Baba, 1994); GU814914, GU814707 (*Paramunida evexa* Macpherson, 1993a); AY351032, AY351201 (*Paramunida labis* Macpherson, 1996); GU814905, GU814696, GU814911, GU814704 (*Paramunida longior* Baba, 1988); GU814886, GU814677 (*Paramunida luminata* Macpherson, 1996); HM060642-4, MT828870-2 (*Paramunida marionis* Cabezas, Macpherson & Machordom, 2010); HM173431-2, MT828868-9 (*Paramunida mozambica* Cabezas, Macpherson & Machordom, 2010); GU814952-53, GU814745-6 (*Paramunida parvispina* Cabezas, Macpherson & Machordom, 2010); EU418006, EU418009, EU417982, EU417985 (*Paramunida salai* Cabezas, Macpherson & Machordom, 2009); GU814898, GU814900, GU814689, GU814691 (*Paramunida setigera* Baba, 1988); GU814877-8, HM173483-4 (*Paramunida tenera* Cabezas, Macpherson & Machordom, 2010); GU814942, GU814735 [*Paramunida tricarinata* (Alcock, 1894)]; GU814862, GU814652, GU814893, GU814684 [*Hendersonida granulata* (Henderson, 1885)]; MT252616, MT25261, MT250542-3 (*Hendersonida parvirostris* Rodríguez-Flores, Macpherson & Machordom, 2020) (Machordom and Macpherson 2004; Cabezas et al. 2012; Rodríguez-Flores et al. 2020). The runs were realized in CIPRES portal (Miller et al. 2010). To estimate the posterior probabilities, four Markov Chains Monte Carlo (MCMC) were run for 1 × 10^7^ generations sampling trees and parameters every 10000 generations. The initial 25 % of the generations were discarded as burn-in. The phylogenetic tree was visualized and edited in FigTree v. 1. 4. 2 (Rambaut 2014); nodes posterior probabilities from the Bayesian inference were included.

**Figure 1.**
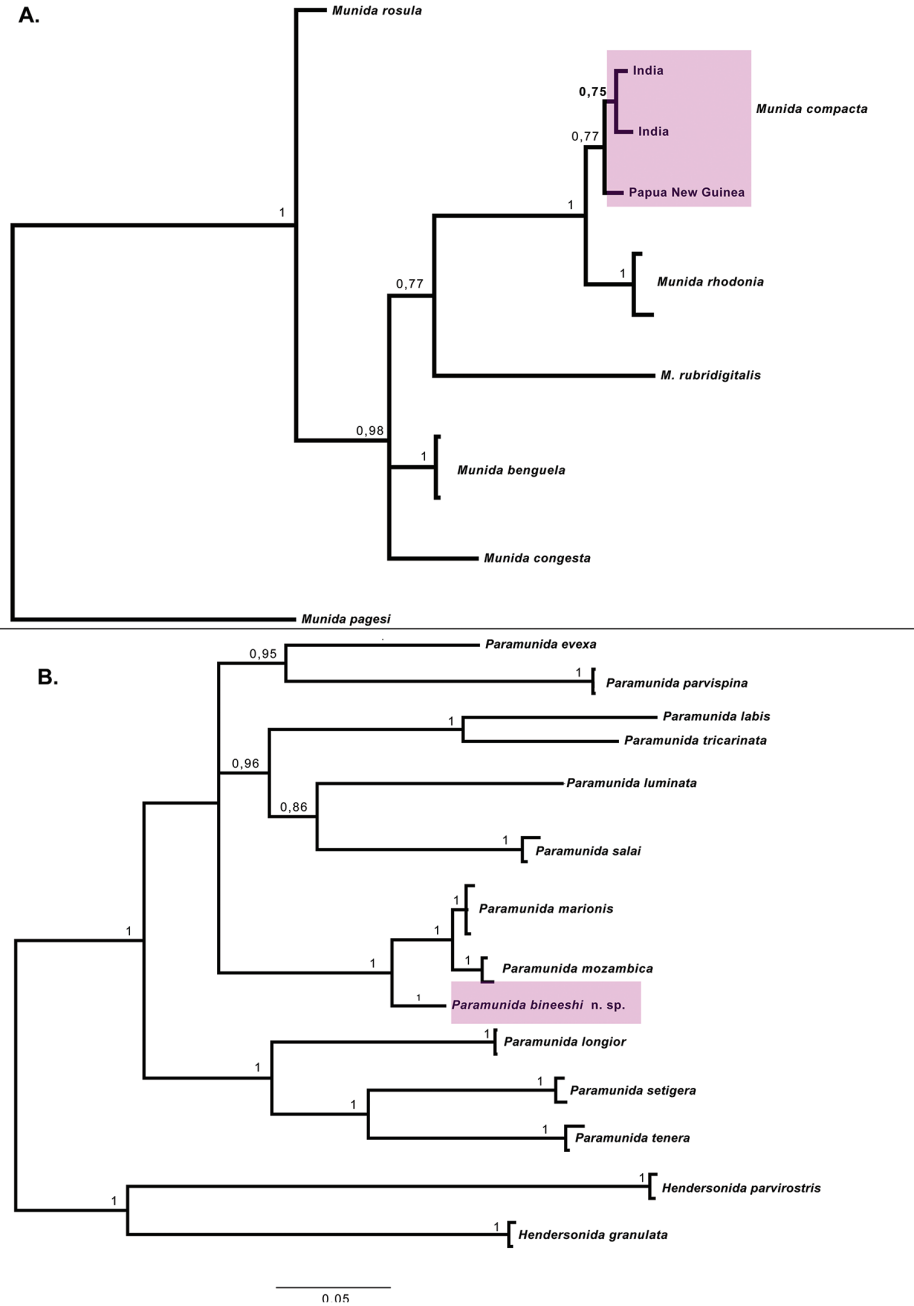
Bayesian phylogenetic relationship between the species found in India and their close relatives. **A** Phylogenetic hypothesis for *Munida compacta***B** phylogenetic hypothesis for *Paramunida bineeshi* sp. nov. and related species. Bayesian support is included above the branches.

## Systematic account

### Family Munididae Ahyong, Baba, Macpherson & Poore, 2010

#### Genus *Agononida* Baba & de Saint Laurent, 1996

##### Agononida prolixa

Taxon classificationAnimaliaDecapoda Munididae

(Alcock, 1894)

AE6C86F7-00F8-5FDC-80D8-4364899D03F1

Figure 2

Munida squamosa var. prolixa Alcock 1894: 322.–Alcock and Anderson 1894: 166; Alcock and Anderson 1895: pl. 13, fig. 3; Alcock 1901: 244; Doflein and Balss 1913: 142; Rao 1974: 302, fig. 1a–c; Macpherson 1993b: 425.
Agononida prolixa .–Ahyong and Poore 2004: 14; Baba 2005: 236; Baba et al. 2008: 51.

###### Material examined.

Male (CL 16.3 mm), Andaman Islands (09°34'21"N, 92°43'94"E; depth 320 m), deepsea trawler, 10 December 2016, K.K. Bineesh leg. (DABFUK/AR-AN-118).

**Figure 2.**
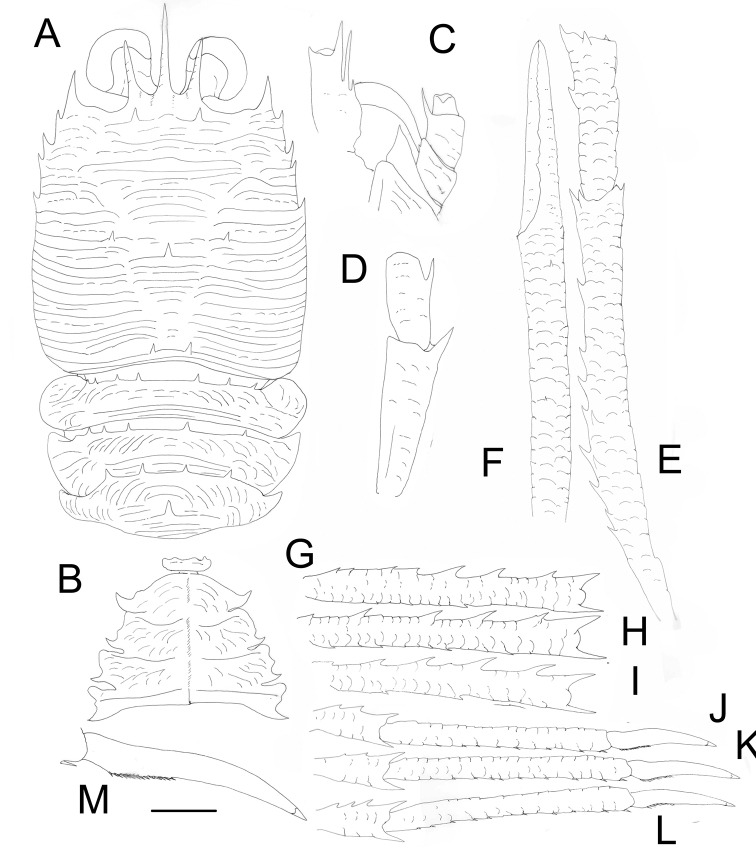
*Agononida prolixa* (Alcock, 1894), male (CL 16.3 mm) (DABFUK/AR-AN-118). **A** Carapace and pleonal tergites 2–4, dorsal **B** sternal plastron **C** cephalic region, showing left antennular and antennal peduncles, ventral **D** right Mxp3, lateral **E** right P1, merus and carpus, lateral **F** right P1, propodus and dactylus, lateral **G** right P2, merus, lateral **H** right P3, merus, lateral **I** right P4, merus, lateral **J** right P2, carpus to dactylus, lateral **K** right P3, carpus to dactylus, lateral **L** right P4, carpus to dactylus, lateral **M** right P2, dactylus, lateral. Scale bars: 4.0 mm (**A, B, E–L**); 2.0 mm (**C, D, M**).

###### Description.

***Carapace***: slightly wider than long. Transverse ridges usually granular, mostly interrupted, with dense, very short, non-iridescent setae; few iridescent setae along lateral margins of carapace. Main transverse striae on posterior part of carapace interrupted in cardiac region. Two epigastric, two postcervical, and one median cardiac spine. Posterior margin with two median spines. Upper orbital margins excavated; lower orbital margins visible dorsally, mesially with spatulate, distally acute process. Lateral margins slightly convex. Anterolateral spine strong, at anterolateral angle, overreaching level of sinus between rostrum and supraocular spines. Second marginal spine anterior to cervical groove well developed. Branchial margins with three spines. Rostrum spiniform, barely half as long as remaining carapace, dorsally convex, distally directed downwards. Supraocular spines slightly thicker than rostral spine, exceeding midlength of rostrum and not reaching end of corneas, slightly divergent, dorsally convex, directed slightly upwards.

***Thoracic sternum***: 0.7× wider than long, sternites with numerous striae. Sternite 3 with median shallow notch, 2.7× wider than long. Sternite 4 0.3× wider than long, with anterior part narrower than sternite 3.

***Pleon***: tergites 2–4 with seven or eight, five, and four spines, respectively, on anterior ridge; tergite 4 with median spine on posterior ridge.

***Eye***: large; maximum corneal diameter 0.3–0.4 distance between bases of anterolateral spines.

***Antennule***: article 1 (distal spines excluded) about one-third CL, slightly overreaching cornea, with two distal spines, mesial spine clearly shorter than lateral spine; two spines on lateral margin, proximal one small, located at midlength of article, distal one long, not overreaching distolateral spine.

***Antenna***: article 1 with one distal spine on mesial margin, reaching end of article 2; article 2 with minute distomesial spine, distolateral angle unarmed; article 3 with long distomesial spine, slightly exceeding article 4.

***Mxp3***: ischium about twice length of merus measured along dorsal margin, distoventrally bearing long spine; merus with one strong median spine on flexor margin; extensor margin unarmed.

***P1***: long, subequal, squamous, 4× CL, with few plumose setae and few scattered iridescent setae on mesial borders of articles. Merus 1.8× CL, 2.6× as long as carpus, with few dorsal and mesial spines. Carpus 0.6× length of palm, nearly 4× as long as broad, with few spines along mesial and dorsal sides. Palm about 7× longer than broad, with few minute dorsal spines. Fingers 0.7× length of palm, unarmed.

***P2*–*4***: moderately long, slender, with numerous plumose setae and few iridescent non-plumose setae along extensor margin of articles. P2 length 3× CL. Meri slightly shorter posteriorly; P2 merus as long as CL, nearly 8× as long as broad, 1.2× longer than P2 propodus; P3 merus 8× longer than broad, 1.2× longer than P3 propodus; P4 merus 7.5× as long as broad, 1.2× longer than P4 propodus. Extensor margins of P2–4 meri with row of six to eight proximally diminishing spines; flexor margins with one strong distal spine followed proximally by several spines and eminences; lateral sides unarmed. Carpi with three or four spines on extensor margin of P2–4; lateral surface unarmed; flexor margin with distal spine. Propodi 11× as long as broad; extensor margin unarmed; flexor margin with 8–10 slender movable spines on P2–4, distal end without fixed spine. Dactyli slender, laterally with longitudinal ridge, 0.5× length of propodi; flexor margin with 12–14 movable spinules, distal half unarmed.

###### Remarks.

*Agononida prolixa* was originally described as Munida squamosa var. prolixa by Alcock (1894) based on the specimens collected by the ‘Investigator’ (Station 115; 11°31'40"N, 92°46'6"E; depth, 344–406 m) from the Andaman Sea (Anonymous 1914). It was subsequently found in the Gulf of Mannar, the Andaman Sea, the Arabian Sea off the coast of Sri Lanka, and south-west of Nicobar Islands (Alcock and Anderson 1894; Alcock 1901; Doflein and Balss 1913). More recently, the species was recorded near Kollam (= Quilon), south-western India at depths of 220–360 m (Rao 1974). The variety described by Alcock (1894) was raised to the rank of species by Ahyong and Poore (2004), which was subsequently followed by Baba (2005) and Baba et al. (2008). *Agononida prolixa* was figured by Alcock and Anderson (1895), and its antennule, antenna, and male pleopod by Rao (1974). We here redescribe the species and illustrate its additional body parts for clarifying its taxonomy.

*Agononida prolixa* belongs to the group of species that have the carapace without protogastric spines, the lateral branchial margin with three spines, the pleonal tergite 4 armed with a spine on the posterior transverse ridge, and the antennal peduncle article 1 with a moderate-sized process that does not overreach article 4. The closest relatives of *A. prolixa* are *A. isabelensis* Cabezas, Macpherson & Machordom, 2009, from the Solomon Islands and Papua New Guinea (Cabezas et al. 2009; Macpherson et al. 2020), and *A. nielbrucei* Vereshchaka, 2005, from New Zealand and the Norfolk Ridge (Vereshchaka 2005; Ahyong 2007). *Agononida prolixa* is easily distinguished from *A. isabelensis* by the presence of a pair of median spines directly anterior to the posterior margin of the carapace, whereas these spines are absent *A. isabelensis*. Differences with *A. prolixa* and *A. nielbrucei* include the following:

- In A. nielbrucei, the dorsal surface of the carapace has one branchiocardiac spine behind the postcervical spine on each side, and a second cardiac spine behind the median large spine. These spines are absent in A. prolixa.
- The distomesial spine of the antennular article 1 is larger than the distolateral spine in A. nielbrucei while it is shorter in A. prolixa.

###### Distribution.

Indian Ocean: Arabian Sea (off Kollam, southwestern India); Gulf of Mannar (off Sri Lanka); and Andaman Sea (Andaman and Nicobar Islands). Depth: 220–752 m.

#### Genus *Munida* Leach, 1820

##### Munida compacta

Taxon classificationAnimaliaDecapoda Munididae

Macpherson, 1997

6D92581B-B33D-5AB5-B769-631DBED9A0DF

Figure 3

Munida compacta Macpherson 1997: 605, fig. 2.–Baba 2005: 261; Baba et al. 2008: 90; Macpherson et al. 2020: 44, figs 5C, 7A.

###### Material examined.

Sakthikulangara Fishing Harbor, Kollam district, Kerala (8°55'30"N, 76°33'22"E; no depth), commercial trawler, T.-Y. Chan leg.: 14 males (CL 12.5–24.5 mm), 8 ovigerous females (CL 15.2–25.9 mm), 4 females (CL 12.9–25.7 mm), 20 March 2017 (DABFUK/AR-AN-119–121); 1 male (CL 22.1 mm), 4 March 2019 (DABFUK/AR-AN-122).

**Figure 3.**
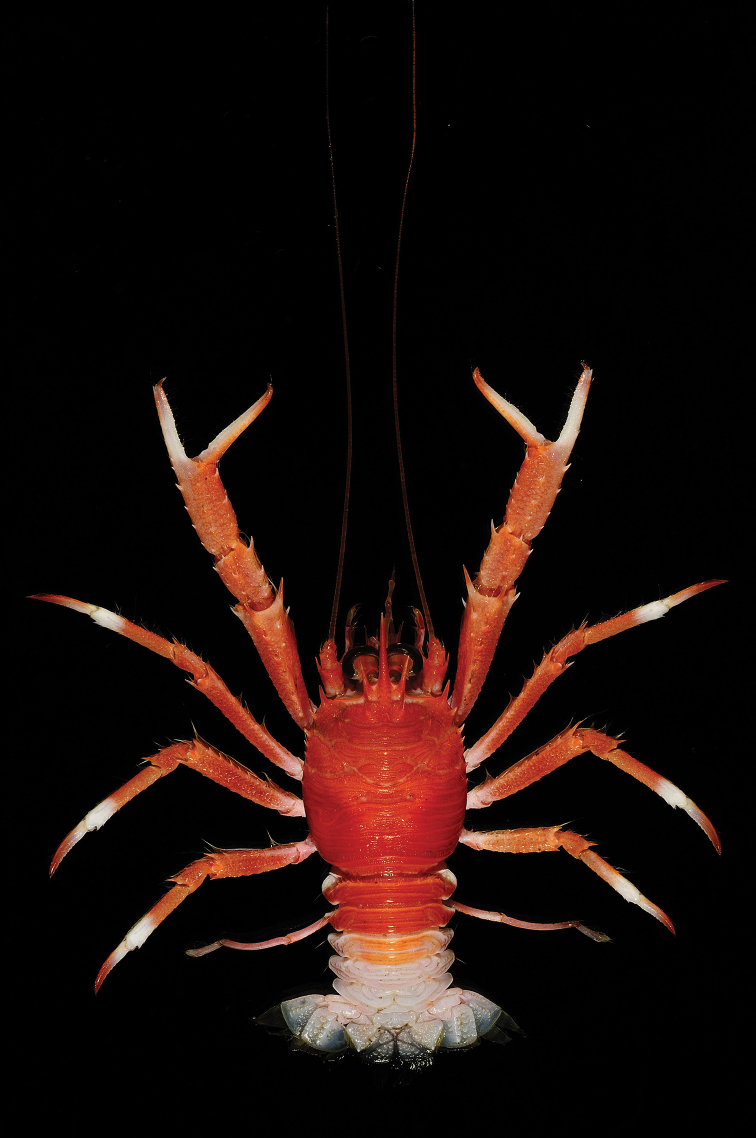
*Munida compacta* Macpherson, 1997, male (CL 22.1 mm) (DABFUK/AR-AN-122), dorsal view.

###### Diagnosis.

Carapace with spiniform rostrum, five spines on branchial margin. Pleonal tergite 2 with more than six spines along anterior ridge, more than five transverse ridges. Thoracic sternum smooth, with few scales on sternite 4; sternite 4 narrowed, subtriangular, narrowly fitting to sternite 3. Eyes large, corneal width distinctly more than one-third distance between base of anterolateral spines. Antennular basal article with distomesial and distolateral spines subequal (or slightly different) in size. Antenna with distomesial spine of article 1 not exceeding end of article 2; distomesial spine of article 2 slightly overreaching end of article 3. Mxp3 merus unarmed on extensor distal margin. P1 fixed finger laterally with (one or two) subterminal spines only, occasionally with proximal spine; movable finger with proximal mesial spine only. P2–4 dactyli with movable spines along entire flexor margin.

###### Colour.

Carapace, pleonal tergites 2 and 3, and appendages reddish or pinkish; pleonal tergites 4–6 and tailfan whitish. Rostrum and supraocular spines reddish. P1 fingers whitish, with reddish tips; distal portion of P2–4 propodi and proximal part of dactyli whitish.

Some specimens from Papua New Guinea have an orange carapace and whitish supraocular spines (Macpherson et al. 2020).

###### Genetic data.

16S and COI.

###### Remarks.

The species was originally described from the specimens collected near the Kei Islands of Indonesia at depths 246–694 m (Macpherson 1997). It was also recently reported from Papua New Guinea at depths 220–1012 m (Macpherson et al. 2020). The specimens from India and Papua New Guinea are morphologically similar except for their colour differences (see notes on the colouration of *M. compacta*). Very low genetic distances were observed among the specimens from south-western India and Papua New Guinea (0.3–0.8% for COI and 0.0–0.6% for 16S), which support they are conspecific (Fig. 1A).

The most morphologically similar species to *M. compacta* is *M. rhodonia* from the south-western Pacific. *Munida compacta* and *M. rhodonia* can be readily distinguished from each other by the size of the second lateral marginal spine of the carapace, which is located immediately behind the anterolateral spine. The second lateral marginal spine of the carapace is well developed in *M. compacta* but very small in *M. rhodonia*. The distal part of the P2–4 propodi is distally broadened in *M. compacta*, while it is uniform in *M. rhodonia*. Furthermore, the ultimate flexor marginal spine (movable) of the P2–4 dactyli is nearly equidistant between the penultimate spine and the tip of the terminal claw in *M. compacta*, whereas it is much closer to the penultimate spine than to the tip of the terminal claw in *M. rhodonia* (Macpherson et al. 2020). A very low genetic divergence was observed between the two species (1.4% for COI and 0.3–0.5% for 16S) (Fig. 1A). In fact, the low genetic distance values may even be within the thresholds considered for intraspecific divergences. However, the morphological differences among the specimens of each species examined from Indonesia, Papua New Guinea, and India are constant (Macpherson 1997; Macpherson et al. 2020; this study), which suggests that *M. compacta* and *M. rhodonia* are separate species. A similar pattern has also been observed in other species comparisons. For instance, a low genetic distance (2.7% for COI, 0.3% for 16S), but with constant morphological differences, was observed between *Munida iris* A. Milne Edwards, 1880, from the West Atlantic and *M. speciosa* von Martens, 1878, from the East Atlantic (Rodríguez-Flores et al. 2019b).

In crustacean studies, a threshold to delimit species has been around 3% genetic divergence for COI (Lefébure et al. 2006; Rodríguez-Flores et al. 2019b). As such, *M. compacta* could be considered a junior subjective synonym of *M. rhodonia*. It is also possible that the sequence data is currently insufficient to differentiate these species. Nei (1987) pointed out that the speciation is based on the patterns of nucleotide substitutions, which occurs at certain rates. These two species seem to constitute lineages in an early stage of speciation. In these situations, further investigations are needed to clarify the taxonomy of *M. compacta*.

*Munida andamanica* Alcock, 1894, which occurs from India to the western Pacific Ocean, is also morphologically similar to *M. compacta*. These species are usually distinguished by the number of transverse ridges on the pleonal tergite 2: *M. compacta* has more than five transverse ridges while *M. andamanica* possesses only up to four transverse ridges (Baba 2005). The specimens of *M. compacta* examined by us, however, possess four to seven transverse ridges, which overlap with the count for *M. andamanica*. Therefore, this character state should be used with caution. Other characters are also variable. For instance, the P1 fixed finger has only one or two subterminal spines on the lateral margin in the specimens from Indonesia and Papua New Guinea, whereas the Indian specimens have both proximal and subterminal spines. Unfortunately, no genetic data is currently available for *M. andamanica*.

The interspecific and intraspecific variations in morphology and colour pattern seen among these three species (*M. andamanica*, *M. compacta*, and *M. rhodonia*) strongly demand a revision of the taxa involving more specimens from different localities, with both morphological and genetic data.

###### Distribution.

Indonesia (Kei Islands); Papua New Guinea; and south-western India. Depth: 220–1012 m.

#### Genus *Paramunida* Baba, 1988

##### Paramunida bineeshi sp. nov.

Taxon classificationAnimaliaDecapoda Munididae

4C3B86B6-D765-551A-A1A2-5C94A930D026

http://zoobank.org/4771CE4C-5FC6-491B-8917-33D5F294F943

Figure 4

###### Type locality.

Andaman Islands (09°34'21"N, 92°43'94"E; depth 320 m).

**T**

###### ype material.

***Holotype***, ovigerous female (CL 12.2 mm), Andaman Islands (09°34'21"N, 92°43'94"E; depth 320 m), deep-sea trawler, 10 December 2016, K.K. Bineesh leg. (DABFUK/AR-AN-123).

**Figure 4.**
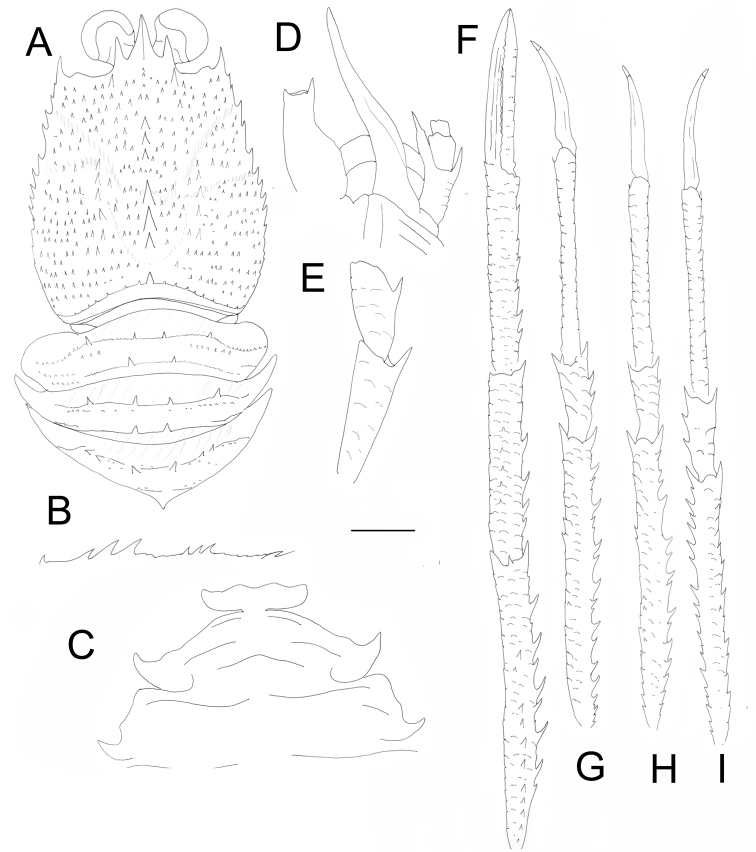
*Paramunida bineeshi* sp. nov., holotype, ovigerous female (CL 12.2 mm) (DABFUK/AR-AN-123). **A** Carapace and pleonal tergites 2–4, dorsal **B** carapace, dorso-lateral **C** sternal plastron **D** cephalic region, showing left antennular and antennal peduncles, ventral **E** right Mxp3, lateral **F** left P1, lateral **G** left P2, lateral **H** left P3, lateral **I** right P4, lateral. Scale bars: 4.0 mm (**A, B, F–I**); 2.0 mm (**C–E**).

###### Description of ovigerous female holotype.

***Carapace***: as long as broad. Dorsal surface covered with numerous spines and spinules, each usually on very short arcuate striae, with few uniramous setae. Epigastric region with two spines, each behind supraocular spine; with median row of spines behind rostral spine. Mesogastric region with three well-developed spines in midline, anterior two spines thicker than anterolateral spine. Cervical groove distinct. Cardiac and anterior branchial regions circumscribed. Cardiac region with median row of three well-developed spines, first thicker than others. Each branchial region with row of moderate-sized spines near cardiac region. Posterior transverse ridge with one well-developed median spine. Frontal margin slightly concave. Lateral margins convex, with small spines. Anterolateral spine reaching sinus between rostral and supraocular spines. Rostrum short, triangular, with thin dorsal longitudinal carina; supraocular spines shorter than rostrum; margin between rostral and supraocular spines straight.

***Thoracic sternum***: thoracic sternite 4 with few arcuate striae; sternites 5 and 6 smooth.

***Pleon***: tergites 2 and 3 each with four moderate-sized spines on anterior ridge, posterior ridge with two moderate-sized median spines. Tergite 4 with four to six spines on anterior ridge; posterior ridge with distinct, single median spine.

***Eyes***: maximum corneal diameter more than one-third distance between bases of anterolateral spines.

***Antennule***: article 1 exceeding cornea, with distomesial spine slightly shorter than distolateral; about twice longer than wide, with fringe of long setae along lateral margin; lateral margin with distal slender portion about half as long as proximal inflated portion.

***Antenna***: anterior prolongation of article 1 clearly overreaching antennular article 1 by about one-third of its length; article 2 (excluding spines) less than twice length of article 3, 1.5× as long as wide, ventral surface with small scales; distomesial spine long, slightly mucronated, slightly exceeding antennal peduncle, nearly reaching midlength of anterior prolongation of article 1, distolateral spine small, not reaching end of article 3; article 3 slightly longer than wide, unarmed.

***Mxp3***: ischium about twice length of merus measured along dorsal margin, distoventrally bearing 1 spine; merus with 1 strong median spine on flexor margin; extensor margin unarmed.

***P1***: long, subequal, squamous, 3.0× CL, with dense plumose setae and scattered iridescent setae on mesial borders of articles. Merus 1.3× CL, 1.7× as long as carpus, with dorsal and mesial spines; distal spines strong, distomesial spine not reaching proximal quarter of carpus. Carpus slightly shorter than palm, 5.5× as long as broad, with spines along mesial and dorsal sides. Palm 6× longer than broad, with spines along mesial margin. Fingers 0.8× length of palm; movable finger with one small proximal mesial spine; fixed finger unarmed.

***P2–4***: long and slender, with scales on lateral sides of meri, carpi, and propodi; each scale with short setae; with dense plumose setae and scattered iridescent setae on extensor borders of articles. P2 2.8× CL, merus 1.2× longer than CL, 10.5× as long as high, 1.5× as long as propodus; propodus 10× as long as high, 1.6× length of dactylus. Merus with well-developed extensor marginal spines, increasing in size distally, flexor margin with few spines and one well-developed distal spine; row of small spines along flexolateral margin. Carpus with small extensor spines, distal spine on extensor and flexor margin. Propodus with small movable flexor spines. Dactylus gently curved, with longitudinal carinae along mesial and lateral sides, ventral border unarmed. P3 with similar spination and segment proportions as in P2; merus as long as P2 merus; propodus and dactylus as long as those of P2. P4 slightly shorter than P2; merus 1.2× CL; propodus and dactylus slightly longer than those of P3; merocarpal articulation clearly exceeding end of anterior prolongation of first segment of antennal peduncle.

###### Etymology.

The new species is named after Kinattum Kara Bineesh who collected this species and kindly passed it to us for study. The species epithet is a noun in the genitive singular.

###### Genetic data.

16S and COI.

###### Remarks.

*Paramunida bineeshi* sp nov. is closely related to *P. mozambica* from the south-western Indian Ocean. The two species can be easily distinguished by the following characters:

- The two anterior spines of the cardiac region are larger than the supraocular spines in the new species, whereas they are smaller in P. mozambica.
- The antennal article 3 is slightly longer than wide in the new species, whereas it is nearly twice as long as wide in P. mozambica.
- The spines along the flexor and extensor margins of P2–4 are larger in the new species than in P. mozambica.
- The genetic divergence between the two species is 4.4–5.0% for COI and 1.0–1.2% for 16S (Fig. 1B).

The new species is also related to *P. marionis* from the southwestern Indian Ocean. The two species can be differentiated on the basis of the following characters:

- The spines of the median row on the gastric and cardiac regions are clearly stronger in the new species than in P. marionis.
- The posterior transverse ridge of the carapace has a well-developed median spine in the new species, whereas the spine is absent in P. marionis.
- The anterolateral spine reaches the sinus between the rostral and supraocular spines in the new species, whereas it clearly extends beyond the sinus in P. marionis.
- The distomesial spine of the antennal article 2 is slightly mucronated or blunt in the new species but spiniform in P. marionis.
- The genetic divergence between the two species is 4.3–4.5% for COI and 0.97% for 16S (Fig. 1B).

###### Distribution.

The new species is only known from the type locality in the Andaman Sea. Depth: 320 m.

## Supplementary Material

XML Treatment for Agononida prolixa

XML Treatment for Munida compacta

XML Treatment for Paramunida bineeshi
